# Restrictions on the use of e-cigarettes in public and private places—current practice and support among adults in Great Britain

**DOI:** 10.1093/eurpub/ckw268

**Published:** 2017-02-04

**Authors:** Leonie S. Brose, Ann McNeill, Deborah Arnott, Hazel Cheeseman

**Affiliations:** 1 Department of Addictions, Institute of Psychiatry, Psychology and Neuroscience, King’s College London, UK and UK Centre for Tobacco and Alcohol Studies (UKCTAS), London, UK; 2 Action on Smoking and Health, 67-68 Hatton Garden, London, EC1N 8JY UK

## Abstract

**Background:**

Debates around policies regulating e-cigarette use make it important to obtain an overview of current practice, people’s attitudes and correlates of policy support. Aims were to assess (i) current practices for e-cigarette use in homes and workplaces; (ii) characteristics associated with allowing e-cigarette use in the home; and (iii) level of, and characteristics associated with, support for extending smoke-free legislation to include e-cigarettes.

**Methods:**

Online survey in 2016, *n* = 11 389 adults in Great Britain. Descriptives for all measures; multivariable logistic regressions assessed correlates of allowing e-cigarette use and support for extension of legislation.

**Results:**

Most (79%) reporting on workplace policies reported some level of restrictions on e-cigarette use. Small majorities would not allow e-cigarette use in their home (58%) and supported an extension of smoke-free legislation (52%; 21% opposed). Allowing use was less likely and supporting an extension more likely among men, respondents from a higher socio-economic status, ex-smokers, never-smokers, non-users of e-cigarettes and respondents with increased perceived harm of e-cigarettes or nicotine (all *P* < 0.001). Older respondents were less likely to allow use and to support an extension and Labour voters more likely to allow use.

**Conclusions:**

In Great Britain, the majority of workplaces has policies restricting e-cigarette use. Over half of adults would not allow use of e-cigarettes in their home and support prohibiting the use of e-cigarettes in smoke-free places. Adjusting for socio-demographics, more restrictive attitudes are more common among never-smokers, never-users and those with increased perception of relative harms of e-cigarettes or nicotine as cause of smoking-related illness.

## Introduction

Worldwide, comprehensive smoke-free legislation is the most widely adopted tobacco control measure.^1^ The United Kingdom (UK) is a country with a high level of tobacco control^2^ that includes smoke-free regulations. Smoking has been prohibited by law in enclosed and substantially enclosed work and public places since July 2007. The law also applies to workplace vehicles used by more than one person at any time and to private cars carrying children under 18 (in place in England, Wales and Scotland, under development in Northern Ireland).^3–5^

Policies banning tobacco smoking in public places or workplaces have been implemented because second-hand smoke exposure is harmful to health.^6^^,^^7^ While the addictive constituent of tobacco smoke is nicotine, the health harms are caused by other constituents of cigarette smoke.^8^^,^^9^ E-cigarettes can be used with a wide range of nicotine concentrations, including no nicotine, but in contrast with traditional cigarettes, e-cigarettes do not contain tobacco, do not create smoke and do not rely on combustion,^10^ leading to much lower levels of harmful constituents in electronic cigarette vapour. There is little evidence that e-cigarette emissions harm the health of bystanders.^10^ E-cigarette use may increase particulate matter in the air,^11^^,^^12^ however, the composition is different from that caused by cigarette smoke^13^ and concentrations are far lower^14^ and sometimes at the same levels as in rooms without smoking or e-cigarette use.^15^ A small number of countries ban all use of e-cigarettes, and some ban use in all enclosed public places or specific places such as workplaces or public transportation,^16^ partly because of fears use may renormalize smoking or undermine enforcement of smoke-free legislation.^17^ In the UK, e-cigarette use in public places is not legislated, but many companies or institutions have drawn up individual policies.^18^^,^^19^ Some guidance on workplace policies is available,^20^ but there is no comprehensive information on current practice and policies.

Historically, public support for tobacco control legislation has been an important component in getting policies adopted in law and effectively implemented.^21^^,^^22^ In the UK, smoke-free policies have been successfully implemented^23^^,^^24^ and are supported by the majority of the public.^4^

Data on support for restricting the use of e-cigarettes in public places have been published from some surveys conducted between 2012 and 2014. They generally found less support among those who had used or tried e-cigarettes and ever-smokers (particularly current smokers), while increased perceptions of harm of e-cigarettes were associated with higher support for restrictions.^25–32^ For smoking, introduction of legislation banning smoking in public places is often associated with a subsequent increase in smoke-free homes,^33–35^ although a recent Canadian study did not find this effect.^36^ Very little evidence is available on current practice around e-cigarette use in homes. One small US survey found 45% of respondents always allowed e-cigarette use in their homes, 23% had some restrictions and 32% never allowed it.^27^ Similar to support for banning use in smoke-free places, restrictions in the home were associated with risk perceptions. Benefits of banning e-cigarette use in the home will differ between homes in which smoking regularly occurs and smoke-free homes. In smoke-free homes, e-cigarette use could increase health harms, whereas in homes with regular smoking, allowing e-cigarette use instead of smoking could have benefits.

As there is much debate around regulations of e-cigarette use, it is important to obtain an overview of current practice, people’s views and to understand the correlates of policy support. The present study therefore aimed to address three research questions:

1. What are current practices for use of e-cigarettes in people’s homes and workplaces?
2. What characteristics are associated with allowing e-cigarette use in the home?
3. What is the level of support for extending smoke-free legislation to include e-cigarette use and what characteristics are associated with support?

## Methods

### Design and procedure

The present findings are based on secondary analyses of data from a cross-sectional online survey carried out in Great Britain between 2 and 23 March 2016. The survey is commissioned annually by the charity Action on Smoking and Health and includes questions relevant to tobacco and e-cigarette policy. The survey used a panel of around 816 000 UK adults (aged 18+) maintained by the market research company YouGov Plc. Panel members were emailed an invitation to participate without information on survey content. Members who agreed were allocated in line with required quotas for age and gender (interlocked), social grade, newspaper readership, ethnicity and region to achieve a good representation of the adult GB population. Members of the panel consent to completing surveys for YouGov in return for a modest financial incentive, and additional ethical approval was not sought due to this pre-existing consent. YouGov abides by British Polling Council and ESOMAR (World Association of Opinion and Marketing Research Professionals) guidelines, maintaining strict participant information confidentiality. All recodes and analyses for the present manuscript were run by the authors using data collected by YouGov.

### Sample

The survey was completed by 12 157 adults. Respondents who had never heard of e-cigarettes (*n* = 518, 4.3%) or selected ‘don’t know’ (*n* = 150, 1.2%) for their e-cigarette use status were excluded because they would not be able to respond to the measures of interest. Additionally, 100 respondents (0.8%) who thought that regular cigarettes were not harmful were excluded, leaving *n* = 11 389 for analyses (93.7%).

### Measures

#### Socio-demographics

Age (18–24; 25–34; 35–44; 45–54; 55 years and over) and gender (male and female) were recorded. Socio-economic status was recorded in two categories: ABC1 that includes managerial, professional and intermediate occupations and C2DE that includes small employers and own account workers, lower supervisory and technical occupations, semi-routine and routine occupations, never workers and long-term unemployed. Respondents who indicated that children under the age of 18 were living at home with them were recorded as living with children. Political orientation was assessed using: ‘If there were a general election held tomorrow, which party would you vote for? Conservative; Labour; UK Independence Party (UKIP); Liberal Democrat; Scottish National Party (SNP) (only for those living in Scotland); Plaid Cymru (only for those living in Wales); some other party; would not vote; don’t know’. Responses were recoded into the three most common responses Conservatives (centre-right party), Labour (centre-left party), UK Independence Party (UKIP, right-wing party), ‘Other party’ and ‘Would not vote/Don’t know’. In general, Conservatives and Labour have supported tobacco control policies, while UKIP is opposed to policies such as plain packaging of tobacco and smoke-free policies in pubs; little information on party lines regarding e-cigarettes is available.

#### Smoking and e-cigarette use

Smoking status was measured using:

Smoking in this survey refers to all burnt tobacco products. It does NOT include e-cigarettes. Which of the following statements BEST applies to you? I have never smoked; I used to smoke but I have given up now; I smoke but I don't smoke every day; I smoke every day.

For logistic regressions, the last two options were combined as ‘current smokers’. E-cigarette use status was measured using:

Which of the following statements BEST applies to you? I have never heard of e-cigarettes and have never tried them [excluded]; I have heard of e-cigarettes but have never tried them; I have tried e-cigarettes but do not use them (anymore); I have tried e-cigarettes and still use them; Don’t know [excluded].

#### Perceptions of nicotine and relative harm

To assess knowledge around nicotine, respondents were asked

According to what you know or believe, what portion of the health risks of smoking comes from nicotine in cigarettes? None or very small; Some but well under half the risk; Around half the risk; Much more than half the risk; Nearly all the risk; Don’t know.

Perceived harm of e-cigarettes relative to cigarettes was measured using:

Do you think electronic cigarettes (e-cigarettes) are more, less or as harmful as regular cigarettes? Electronic cigarettes are A LOT MORE harmful than regular cigarettes; Electronic cigarettes are MORE harmful than regular cigarettes; Electronic cigarettes are JUST AS harmful as regular cigarettes; Electronic cigarettes are LESS harmful than regular cigarettes; Electronic cigarettes are A LOT LESS harmful than regular cigarettes; Electronic cigarettes are completely harmless; Don’t know; Not applicable – I do not think regular cigarettes are harmful [excluded].

For logistic regressions, responses were collapsed into more harmful/just as harmful, less harmful, don’t know.

#### Current practice and policy support

Current practice around e-cigarette use in the home was assessed using: “If someone wanted to use an electronic cigarette in your home would you allow them to? Yes, I would; No, I would not; Don't know.” All respondents were asked about smoking in the home: “Does anyone smoke in your home most days? Yes; No; Don’t know.” Respondents were asked about e-cigarette policies in their workplace: “Are people allowed to use electronic cigarettes in your workplace? Yes, anywhere with no restrictions; Yes, indoors but with restrictions; Yes, but only outdoors; No, they are not allowed anywhere; Don't know; Not applicable”. Support for extending smoke-free legislations to e-cigarette use was assessed:

It is against the law to smoke in enclosed public places and workplaces, but because electronic cigarettes do not produce smoke they are not included in this legislation. How strongly, if at all, do you support or oppose extending this law to cover the use of electronic cigarettes?

Responses were given on a five-point scale from ‘strongly support’ to ‘strongly oppose’ or an additional ‘don’t know’ option. For logistic regressions, this was collapsed into support (“strongly support” and “tend to support”) vs. all other responses.

### Analysis

Percentages were used to describe socio-demographics, smoking status, e-cigarette use status, perceptions of nicotine and relative harm, current practices in workplaces (overall and by socio-economic status) and homes and support for extending smoke-free laws.

Logistic regressions were used to assess the association between allowing e-cigarette use in the home and socio-demographics, smoking status, e-cigarette use status, perceptions of nicotine and relative harm and living with children. Assessment of bivariate associations was followed by multivariable models including all variables. Similarly, logistic regressions assessed associations between support for extending smoke-free laws and socio-demographics, smoking status, e-cigarette use status, perceptions of nicotine and relative harm and living with children. Multicollinearity was checked using the same variables in multiple linear regressions. When including smoking in the home in multivariable logistic regressions for allowing e-cigarette use in the home, multicollinearity was indicated and therefore only the bivariate association is reported.

## Results

### Descriptives

Table 1 shows information about the respondents. Compared with national statistics from 2015,^37^ the present sample was older; specifically, a smaller proportion of the present sample was aged 25–34 (8.5 vs. 17.2%) and a larger proportion was aged 55 or over (49.2 vs. 37.2%). Higher social grades were somewhat overrepresented compared with 2011 figures for Great Britain where 53% were in grades ABC1.^38^ This may to some extent explain the comparatively low smoking prevalence; another representative survey found it to be around 18% in 2016.^39^

**Table 1:** Sample description, *N* = 11 389

	%		%
Gender		E-cigarette use status
Female	52.8	Never tried	84.3
Male	47.2	Tried/used but not using	9.9
Age		Current user	5.8
18–24	9.3	Perceived relative harm of e-cigarettes	
25–34	8.5	A lot more harmful [than cigarettes]	1.2
35–44	14.1	More harmful	1.4
45–54	18.8	Just as harmful	22.4
55+	49.2	Less harmful	30.1
Social grade		A lot less harmful	14.9
ABC1	62.1	E-cigarettes are completely harmless	1.2
C2DE	37.9	Don’t know	28.8
Living with children under 18 years		Risks of smoking from nicotine	
Yes	18.4	None or very small	7.9
No	81.6	Some but well under half	19.9
Political orientation		Around half	17.6
Labour	28.7	Much more than half	19.2
Conservative	25.7	Nearly all	15.1
UKIP	13.6	Don’t know	20.3
Other party	12.7	Someone smokes in home most days	
Would not vote/don’t know	19.3	Yes	12.4
Smoking status		No	87.3
Never smoker	49.2	Don’t know	0.3
Ex-smoker	36.6		
Non-daily smoker	3.5		
Daily smoker	10.8		

### Current practice in homes and workplaces

**Figure 1:**
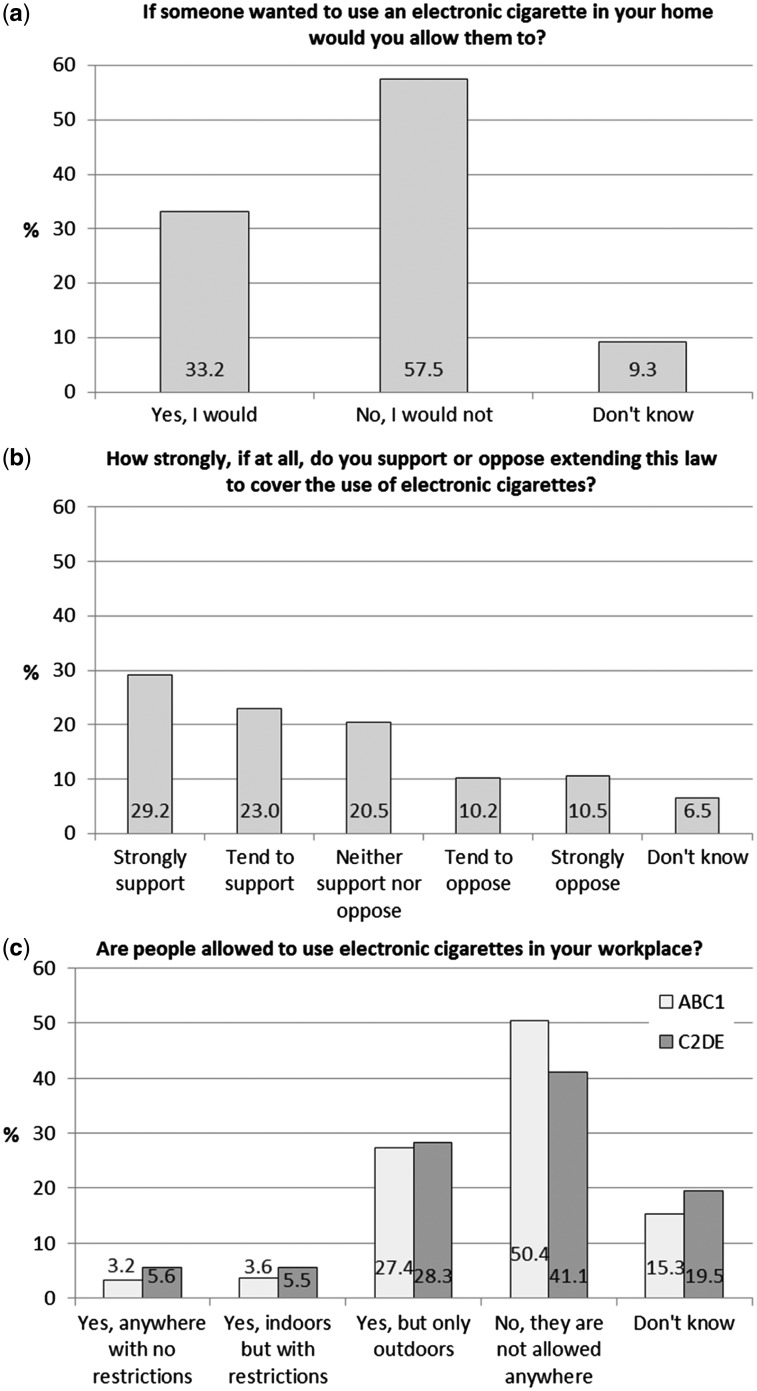
(a) E-cigarette use in the workplace by socio-economic status, *n* = 6798 (excluding ‘not applicable’); (b) Allowing e-cigarette use in the home by socio-economic status, *N* = 11 389; (c) Support for extending smoke-free laws, *N* = 11 389. Full text of question: “It is against the law to smoke in enclosed public places and workplaces, but because electronic cigarettes do not produce smoke they are not included in this legislation. How strongly, if at all, do you support or oppose extending this law to cover the use of electronic cigarettes?”

Workplace policies were not applicable to *n* = 4591 (40.3%) respondents, presumably often because they did not work outside the home. Of those to whom it was applicable, 4.1% reported no restrictions on e-cigarette use, 4.3% reported use was allowed indoors with restrictions, 27.6% reported use was allowed outdoors, 47.3% reported use was not allowed anywhere and 16.8% did not know their workplace policy. Respondents with a higher socio-economic status were more likely to report restrictions (χ^2^ = 90.26, *P* < 0.001; figure 1a).

A small majority (57.5%) would not allow e-cigarette use in their home (figure 1b). In adjusted analysis, all characteristics were associated with allowing use. Allowing use was far less likely among those who had never used e-cigarettes, or perceived e-cigarettes as at least as harmful as cigarettes (table 2). Men, older respondents, those of a higher socio-economic status, those voting Conservative, other party or undecided/non-voters, ex-smokers and never smokers, those who had tried but were not using e-cigarettes and those who perceived nicotine to be causing more than a little of the health harms of smoking were all less likely to allow e-cigarette use in their home. No multicollinearity was indicated if smoking in the home was not included (VIF from 1.02 to1.36, tolerance 0.74 to 0.98).

**Table 2:** Associations with allowing e-cigarette use in the home, logistic regressions, *N* = 11 389

	Would allow use in home, %	Unadjusted/Bivariate		Adjusted/Multivariable	
		OR	95% Confidence interval	OR	95% Confidence interval
Gender					
Male (referent)	32.4	1		1	
Female	33.9	1.07	0.99–1.16	1.33	1.21–1.47
Age					
18–24 (referent)	42.5	1		1	
25–34	37.1	0.80	0.67–0.96	0.91	0.73–1.13
35–44	34.1	0.70	0.60–0.82	0.91	0.74–1.12
45–54	33.8	0.70	0.60–0.81	0.87	0.72–1.05
55 and over	30.3	0.59	0.51–0.67	0.73	0.61–0.87
Social grade					
C2DE (referent)	37.7	1		1	
ABC1	30.4	0.72	0.67–0.78	0.80	0.73–0.89
Living with children					
No	34.1	1		1	
Yes	29.2	0.78	0.72–0.88	0.66	0.58–0.76
Political orientation					
Labour (referent)	38.6	1		1	
Conservative	27.4	0.60	0.54–0.67	0.71	0.63–0.81
UKIP	36.4	0.91	0.81–1.03	0.93	0.80–1.09
Other	33.7	0.81	0.71–0.92	0.81	0.69–0.95
Would not vote/don’t know	30.4	0.70	0.62–0.78	0.73	0.64–0.84
Smoking status					
Current smoker (referent)	76.9	1		1	
Ex-smoker	33.9	0.15	0.14–0.18	0.26	0.22–0.30
Never smoker	20.0	0.08	0.07–0.09	0.16	0.14–0.19
E-cigarette use status					
Current user (referent)	91.5	1		1	
Tried/used but not using	78.2	0.33	0.25–0.45	0.38	0.27–0.52
Never tried	23.9	0.03	0.02–0.04	0.09	0.07–0.12
Risks of smoking from nicotine					
None/very small (referent)	62.6	1		1	
Some/around half	38.6	0.38	0.32–0.44	0.58	0.49–0.69
Much more than half/nearly all	23.4	0.18	0.16–0.21	0.44	0.36–0.53
Don’t know	28.4	0.24	0.20–0.28	0.46	0.38–0.56
Perceived relative harm					
Less harmful (referent)	47.3	1		1	
More harmful/just as harmful	15.5	0.21	0.18–0.23	0.22	0.19–0.25
Don’t know	26.0	0.39	0.36–0.43	0.47	0.42–0.53
Someone smokes in home most days					
Yes	82.0	1			
No/don’t know	26.3	0.08	0.07–0.09		

### Policy support

Just over half (52.2%) supported an extension of smoke-free laws to include the use of e-cigarettes and a fifth (20.7%) were opposed to an extension (figure 1c).

Several characteristics were associated with support for extending smoke-free laws in adjusted analysis (table 3). Men, younger adults, those from higher social grades were more likely to support an extension. Those who would vote Labour were more likely to support an extension than UKIP voters and those who would not vote or were undecided. Never-smokers and ex-smokers were more likely to support an extension than current smokers. Those who had never tried e-cigarettes were more likely to support an extension than current e-cigarette users. Compared with those who knew that only a small part of the health risks of smoking is due to nicotine, all other responses to this question were associated with increased likelihood of supporting an extension and odds increased with the level of risk ascribed to nicotine. In line with this, those who perceived e-cigarettes to be at least as harmful as cigarettes were more supportive of an extension of the law (table 3). No multicollinearity was indicated (VIF from 1.02 to 1.36, tolerance 0.74 to 0.98).

**Table 3:** Associations with support for extending smoke-free laws to include e-cigarettes, logistic regressions, *N* = 11 389

	Support extending laws, %	Unadjusted/Bivariate		Adjusted/Multivariable	
		OR	95% Confidence interval	OR	95% Confidence interval
Gender					
Male (referent)	52.7	1		1	
Female	51.9	0.97	0.90–1.04	0.82	0.76–0.89
Age					
18–24 (referent)	53.2	1		1	
25–34	53.0	0.99	0.84–1.18	0.90	0.74–1.10
35–44	52.1	0.96	0.82–1.12	0.80	0.67–0.96
45–54	51.7	0.94	0.81–1.09	0.81	0.68–0.96
55 and over	52.2	0.96	0.84–1.10	0.79	0.68–0.93
Social grade					
C2DE (referent)	48.0	1		1	
ABC1	54.9	1.32	1.22–1.42	1.25	1.15–1.37
Living with children					
No	51.7	1		1	
Yes	54.6	1.12	1.02–1.23	1.15	1.02–1.29
Political orientation					
Labour (referent)	52.2	1		1	
Conservative	56.9	1.21	1.09–1.34	1.06	0.95–1.18
UKIP	45.7	0.77	0.68–0.87	0.73	0.64–0.84
Other	56.1	1.17	1.03–1.32	1.10	0.96–1.26
Would not vote/don’t know	48.3	0.86	0.77–0.95	0.82	0.73–0.92
Smoking status					
Current smoker (referent)	26.0	1		1	
Ex-smoker	51.1	2.97	2.62–3.37	2.11	1.82–2.44
Never smoker	60.8	4.41	3.90–4.99	2.67	2.30–3.11
E-cigarette use status					
Current user (referent)	20.1	1		1	
Tried/used but not using	27.7	1.53	1.21–1.93	1.26	0.99–1.61
Never tried	57.4	5.36	4.41–6.52	2.50	2.02–3.09
Risks of smoking from nicotine					
None/very small (referent)	32.6	1		1	
Some/around half	52.1	2.25	1.93–2.62	1.67	1.42–1.97
Much more than half/nearly all	61.1	3.24	2.78–3.78	2.06	1.73– 2.44
Don’t know	45.2	1.70	1.45–2.00	1.28	1.07–1.54
Perceived relative harm					
Less harmful (referent)	44.7	1		1	
More harmful/just as harmful	75.6	3.84	3.47–4.25	3.53	3.17– 3.93
Don’t know	44.1	0.98	0.89–1.07	0.92	0.83–1.01

## Discussion

This survey in Great Britain indicates that over half of adults would not allow use of e-cigarettes in their home. Allowing e-cigarette use in the home was associated with decreased perceptions of harm, smoking and knowing that nicotine is not the main cause of health risks of smoking, but the strongest associations were with having tried an e-cigarette and living in a home where someone smoked most days. Notably, even among those living in homes with someone smoking most days, almost a fifth would not allow e-cigarette use. The survey findings also indicate that restrictions on e-cigarette use in workplaces are common. A considerable proportion did not know the regulations regarding e-cigarettes at their workplace; this may be because workplaces may not have a policy or because respondents never had reason to find out. About half of those describing workplace policies reported that e-cigarette use was not allowed anywhere. This may be inflated because offices may not have outdoor spaces perceived to be part of the workplace. Just over half of adults were supportive of extending current smoke-free laws to include vaping. Never smokers and ex-smokers, representing the majority of the British adult population, were more likely to support a ban on the use of e-cigarettes in smoke-free places than current smokers. Knowledge about nicotine and more realistic perceptions of harm of e-cigarettes relative to tobacco cigarettes were associated with less support for extending restrictions. However, even among those with the lowest harm perceptions and accurate nicotine knowledge, considerable proportions were supportive, indicating other factors also drive support for this policy.

The survey could not assess all factors that may influence policy support or rules on e-cigarette use in the home. Other limitations include that the wording of the question assessing policy support was complex and had a low reading ease (high reading age: Flesch-Kincaid grade level 12.9, SMOG index 15.4.^40^ However, the question is very close in its wording to a long-standing question assessing support for smoke-free legislation which has been used to assess changes over time. Although the recruitment aimed to ensure representativeness of the British adult population, the present sample differed from the general population in some characteristics. Over-represented groups were more likely not to allow e-cigarette use in the home and more likely to support an extension of smoke-free laws, suggesting that the GB population as a whole may be slightly more liberal in their views on e-cigarettes than the present sample.

The questions on perceived harm of e-cigarettes did not differentiate between harm to the user or bystander or between e-cigarettes containing nicotine and those not containing nicotine. Perception of harm may differ with these characteristics and more detailed measurement of harm perception could be of interest.

The existing smoke-free legislation enjoys wider support than a possible extension to include e-cigarettes. In 2014, the most recent available data, 82% of the population supported the existing smoke-free legislation^4^ with 8% opposing it, compared with 51% supporting restricting e-cigarette use in the same way and 21% opposing it. Support for smoke-free legislation has increased over time; however, over two-thirds of the population were in favour before the legislation was implemented.^4^

The present survey extends previous evidence on support for e-cigarette policies by including political affiliation and never-smokers in a large sample of the population in a country with a strong tobacco control profile. As might be expected, with the inclusion of never-smokers, overall support for restricting e-cigarette use in smoke-free places was slightly higher than in a recent survey of ex-smokers and smokers.^32^ As expected, affiliation with a party opposed to tobacco control policies was associated with reduced support for e-cigarette regulation. Across countries and using different measures, surveys consistently find less support for restricting e-cigarette use among those who have experience with e-cigarettes or accurately perceive them to be less harmful than smoking.^27^^,^^29^

Future research should assess support over time and in countries with different e-cigarette and smoke-free legislation, in addition to tracking changes in perceived relative harm and use of e-cigarettes by smokers to monitor potential unintended consequences. This should include tracking before and after changes in legislation regulating the use of e-cigarettes in public places.

## Conclusion

In Great Britain, the majority of workplaces has policies restricting the use of e-cigarettes. Half of adults would not allow use of e-cigarettes in their home and support prohibiting the use of e-cigarettes in smoke-free places. More restrictive attitudes are more common among never-smokers, never-users of e-cigarettes and those with increased perception of relative harms of e-cigarettes compared with cigarettes or nicotine as cause of smoking-related illness.
